# Cocoa intake and arterial stiffness in subjects with cardiovascular risk factors

**DOI:** 10.1186/1475-2891-11-8

**Published:** 2012-02-10

**Authors:** José Ignacio Recio-Rodríguez, Manuel A Gómez-Marcos, María C Patino-Alonso, Cristina Agudo-Conde, Emiliano Rodríguez-Sánchez, Luis García-Ortiz

**Affiliations:** 1 Primary Care Research Unit, La Alamedilla Health Center, Avda. Comuneros 27, 37003 Salamanca, Spain; 2 Statistics Department, University of Salamanca, Salamanca, Spain

**Keywords:** Cocoa, Atherosclerosis, Blood pressure

## Abstract

**Background:**

To analyze the relationship of cocoa intake to central and peripheral blood pressure, arterial stiffness, and carotid intima-media thickness in subjects with some cardiovascular risk factor.

**Findings:**

Design: A cross-sectional study of 351 subjects (mean age 54.76 years, 62.4% males). Measurements: Intake of cocoa and other foods using a food frequency questionnaire, central and peripheral (ambulatory and office) blood pressure, central and peripheral augmentation index, pulse wave velocity, ambulatory arterial stiffness index, carotid intima-media thickness, and ankle-brachial index.

Results: Higher pulse wave velocity and greater cardiovascular risk were found in non-cocoa consumers as compared to high consumers (*p *< 0.05). In a multivariate analysis, these differences disappeared after adjusting for age, gender, the presence of diabetes, systolic blood pressure and antihypertensive and lipid-lowering drug use. All other arterial stiffness measures (central and peripheral augmentation index, ambulatory arterial stiffness index, ankle-brachial index, and carotid intima-media thickness) showed no differences between the different consumption groups.

**Conclusions:**

In subjects with some cardiovascular risk factors, cocoa consumption does not imply improvement in the arterial stiffness values.

**Trial Registration:**

Clinical Trials.gov Identifier: NCT01325064.

## Introduction

Cocoa intake is related to a lower cardiovascular risk [1,2]. Although, two recently published metaanalyses coincide that it is unclear whether cocoa consumption is related to reductions in adverse cardiovascular outcomes [3,4].

The benefits of cocoa could be explained by an improved endothelial function and a beneficial effect on blood pressure [5], particularly in subjects with high levels of blood pressure [6]. However, the relationship of usual cocoa consumption to arterial stiffness parameters and central blood pressure is not clear [7,8], although cocoa intake has been related to a lower prevalence of atherosclerotic plaques in carotid arteries [9,10]. The relationship between cocoa consumption and arterial stiffness has only been studied in healthy individuals without cardiovascular risk factors.

The purpose of this study was to analyze the relationship of cocoa intake to central and peripheral blood pressure, arterial stiffness, and carotid intima-media thickness in subjects with some cardiovascular risk factor.

## Methods

A cross-sectional study conducted on 351 subjects in a primary care setting. Participants were recruited at primary care offices during 2010. Sample size was estimated to detect a minimum difference of 1 m/sec in pulse wave velocity (PWV) between two of the three groups in which sample was divided based on cocoa intake. Assuming an alpha risk of 0.05 and a beta risk of 0.2 in a two-sided test and a standard deviation of 2.1, 93 subjects per group were required, 279 in total. The study adhered to the principles of the Declaration of Helsinki [11], and was approved by an independent ethics committee of Salamanca University Hospital (Spain). Written informed consent to participate in the study was obtained from all subjects.

Information about the frequency of cocoa intake and all other foods was collected using the food frequency questionnaire of the University of Navarre, validated for Spain [12]. This questionnaire categorizes cocoa consumption as follows: Never, 1-3 servings/month, weekly, 2-4 servings/week, 5-6 servings/week, daily, 2-3 servings/day, 4-6 servings/day, more than 6 servings/day. Cocoa intake was determined as follows: the average daily consumption (weight) of cocoa-containing foods and beverages was multiplied by their individual percentage of cocoa contents, which were derived from food labels and from published data. Cocoa intake from individual foods was summed to yield the actual intake of cocoa in grams per day for each subject. We posteriorly divided the subjects according to estimated cocoa consumption. Low cocoa consumption was taken to represent the intake of ≤ 1 serving/week.

Procedures for measuring office and ambulatory blood pressure, ankle-brachial index (ABI), body mass index, and abdominal waist circumference have been reported in a prior publication [13].

Central blood pressure (CBP) and central and peripheral augmentation index (CAIx, PAIx) were estimated using the SphygmoCor System (AtCor Medical Pty Ltd., Head Office, West Ryde, Australia). With the patient sitting and the arm resting on a rigid surface, pulse wave in radial artery was tested and used to estimate the aortic pulse wave using a mathematic transformation. Inter-observer reliability was assessed before the start of the study using an intraclass correlation of 0.974 (95% CI: 0.936-0.989) in repeated measures in 22 subjects and with Bland-Altman analysis, where inter-observer agreement limits were 0.454 (95% CI: -9.876-10.785). Using the SphygmoCor System (Vx pulse wave velocity), PWV was measured with the patient in the supine position, estimating the delay in pulse wave at carotid and femoral level as compared to the electrocardiogram wave. Ambulatory arterial stiffness index (AASI) is defined as 1 minus the regression slope of 24-hour diastolic on systolic blood pressure [14].

Carotid ultrasonography to assess intima-media thickness of the common carotid artery (C-IMT) was performed by two investigators specifically trained for that purpose. A Sonosite Micromax ultrasound device (Sonosite Inc., Bothell, Washington, USA) paired with a 5-10 MHz multifrequency high-resolution linear transducer with Sonocal software was used for performing automatic measurements of IMT, in order to optimize reproducibility. Reliability of this measurement was evaluated before study start using intraclass correlation coefficient, which showed values of 0.974 (95% CI: 0.935-0.990) for intra-observer agreement on repeated measurements in 20 subjects, and 0.897 (95% CI: 0.740-0.959) for inter-observer agreement. Six measurements were taken on each carotid artery, using average values (average IMT) and maximum values (maximum IMT) calculated by the software automatically. Measurements were taken following the recommendations of the Manheim Carotid Intima-Media Thickness Consensus [15]. Cardiovascular risk was estimated using the SCORE [16] and D'Agostino [17] equations.

Continuous variables are expressed as the mean ± standard deviation, and qualitative variables as frequency distributions. Association between qualitative variables was assessed using a Chi-square test, differences in means between groups with an ANOVA test, and a Scheffé test was used as a post hoc test with an alpha value < 0.05. We used a multivariate analysis based on the ANCOVA method (analysis of covariance), to compare the arterial stiffness variables between different cocoa-intake groups using as adjustment variables age, gender, systolic blood pressure, diabetes and the presence of medical treatment (antihypertensive and lipid-lowering drugs). SPSS/PC + version 18.0 statistical software was used (SPSS Inc., Chicago, Illinois, USA).

## Results

Sample consisted of 351 subjects (mean age 54.76 years, 62.4% males). Mean cocoa consumption of the global sample was 4.30 ± 9.08 g/day, with an intake among those who consumed some cocoa of 7.74 ± 11.04 g/day, and a median value of 2 g/day. Table 1 shows the clinical and demographic characteristics of the population, as well as measures of arterial stiffness, central and peripheral blood pressure, C-IMT, and consumption of energy and immediate principles. Non-cocoa consumers had an older mean age and a lower 24-hour diastolic blood pressure as compared to the other groups, and also lower proportions of diabetic and dyslipidemic patients (*p *< 0.05).

**Table 1:** General demographic and clinical characteristics

	Non-consumption (n = 156)	Low consumption ≤ 1 serving/week (n = 106)	High consumption > 1 serving/week (n = 89)	*p*
Age	57.32 ± 11.10	54.11 ± 11.76	51.07 ± 11.57	< 0.001

Male (n, %)	93 (59.60)	59 (55.70)	67 (75.30)	0.012

Female (n,%)	63 (40.40)	47(44.30)	22 (24.70)	-

Diabetes (n, %)	58 (37.20)	23 (21.70)	8 (9.00)	< 0.001

Hypertension (n, %)	126 (80.77)	83 (53.21)	64 (41.03)	0.273

Obesity (BMI > 30) (n, %)	58 (37.20)	36 (34.00)	20 (22.50)	0.057

Dyslipidemia (n, %)	109 (69.90)	84 (79.20)	75 (84.30)	0.034

Smoking (n, %)	35 (22.40)	27 (25.50)	23 (25.80)	0.783

Antihypertensive drugs (n, %)	84 (53.80)	48 (45.30)	33 (37.10)	0.037

Lipid lowering drugs (n, %)	59 (37.80)	27 (25.50)	14 (15.70)	0.001

BMI (Kg/m^2^)	28.78 ± 4.61	28.46 ± 4.31	27.50 ± 3.74	0.082

Waist circumference (cm.)	98.21 ± 12.74	96.83 ± 11.44	95.93 ± 11.74	0.342

Serum glucose (mg/dL)	106.12 ± 36.32	95.42 ± 25.89	89.52 ± 15.74	< 0.001

Total cholesterol (mg/dL)	200.29 ± 40.16	205.41 ± 35.33	206.07 ± 34.59	0.400

Cardiovascular risk (Score)	5.08 ± 6.12	4.16 ± 7.06	2.82 ± 4.39	0.020

Cardiovascular risk (D'Agostino)	22.15 ± 15.60	18.68 ± 18.12	16.23 ± 13.66	0.017

Office SBP (mmHg)	138.81 ± 17.17	138.78 ± 17.86	140.12 ± 16.62	0.822

Office DBP (mmHg)	85.89 ± 10.74	87.09 ± 10.39	87.87 ± 11.27	0.357

Peripheral pulse pressure (mmHg)	52.91 ± 13.00	51.68 ± 13.01	52.24 ± 11.45	0.736

Central SBP (mmHg)	131.66 ± 18.35	130.58 ± 17.26	132.17 ± 19.09	0.820

Central DBP (mmHg)	87.87 ± 11.50	88.05 ± 10.78	89.49 ± 11.73	0.534

Central Pulse pressure (mmHg)	43.78 ± 14.12	42.53 ± 11.54	42.67 ± 12.76	0.696

24 h SBP (mmHg)	124.12 ± 12.48	125.73 ± 12.64	127.78 ± 12.39	0.087

24 h DBP (mmHg)	75.51 ± 9.52	77.47 ± 9.91	79.20 ± 9.35	0.014

24 h Pulse pressure (mmHg)	48.60 ± 9.70	48.26 ± 9.12	48.58 ± 9.26	0.955

PWV (m/s)	9.22 ± 2.15	8.93 ± 2.41	8.47 ± 1.96	0.039

C-IMT mean (mm.)	0.74 ± 0.10	0.72 ± 0.11	0.71 ± 0.13	0.109

C-IMT maximum (mm.)	0.91 ± 0.12	0.89 ± 0.13	0.88 ± 0.16	0.100

CAIx	30.24 ± 11.25	30.84 ± 11.34	28.81 ± 11.11	0.439

PAIx	94.74 ± 22.91	92.03 ± 17.95	88.43 ± 21.39	0.085

AASI	0.38 ± 0.06	0.37 ± 0.05	0.37 ± 0.05	0.331

Day AASI	0.38 ± 0.06	0.37 ± 0.05	0.37 ± 0.05	0.363

Night AASI	0.39 ± 0.14	0.37 ± 0.16	0.39 ± 0.16	0.786

ABI	1.07 ± 0.10	1.08 ± 0.11	1.08 ± 0.09	0.791

Total energy (Kcal/day)	2312.80 ± 691.41	2487.15 ± 660.39	2907.62 ± 777.29	< 0.001

Carbohydrates (g/day)	259.47 ± 95.26	269.97 ± 82.01	323.36 ± 99.28	< 0.001

Protein (g/day)	101.87 ± 32.40	108.73 ± 30.33	113.29 ± 28.44	0.016

Total fat (g/day)	87.35 ± 31.93	99.60 ± 36.93	120.11 ± 37.92	< 0.001

BMI: Body mass index, SBP: Systolic blood pressure, DBP: Diastolic blood pressure, IMT: Intima-media thickness, PWV: Pulse wave velocity, C-IMT: Carotid intima-media thickness, CAIx: Central augmentation index, PAIx: Peripheral augmentation index, AASI: Ambulatory arterial stiffness index, ABI: ankle-brachial index

Data for qualitative variables are expressed as n (%) and quantitative variables as mean ± standard deviation

*p*: statistically significant differences (*p *< 0.05)

The proportion of patients treated with drugs was greater among the non-consumers of cocoa than in the high consumption group, referred to both the antihypertensive agents (53.80% vs 37.80%) and the lipid-lowering drugs (37.10% vs 15.70%). A higher proportion of males (30.59%) as compared to females (16.67%) reported high cocoa consumption.

**Figure 1:**
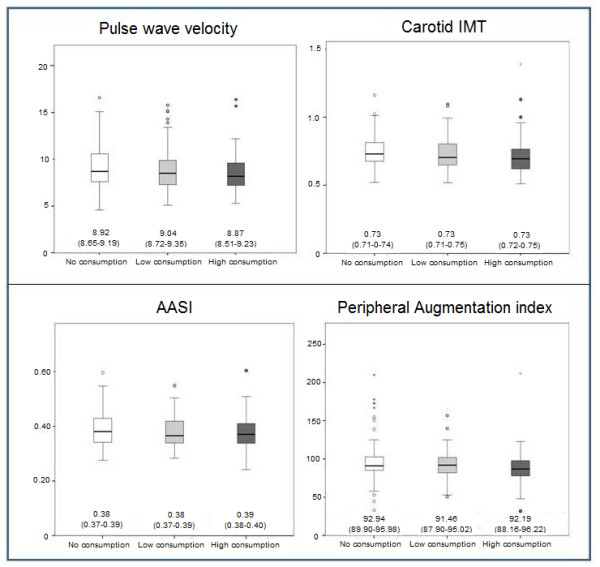
**Multivariate analysis (ANCOVA)**. Box plots of PWV, AASI, C-IMT, and PAIx in the different cocoa consumption groups. Mean (95% CI) adjusted for age, gender, the presence of diabetes, systolic blood pressure and antihypertensive and lipid-lowering drug use.

PWV showed a significant difference between the non-consumers (9.22 m/sec) and the high consumption group (8.47 m/sec) (*p *= 0.039). However, in the multivariate analysis, on adjusting for age, gender, the presence of diabetes, systolic blood pressure and antihypertensive and lipid-lowering drug use, this difference disappeared. The power of the contrast was 81% for detecting differences in PWV between high consumers and non-consumers, and 42% between high consumers and low consumers. All other arterial stiffness measures showed no differences between the different consumption groups (Figure 1).

We re-analyzed the data, excluding the diabetics, without any modification of the results or conclusions obtained.

## Discussion

Although there was a trend to a decreased arterial stiffness as cocoa consumption increased, data collected show that cocoa intake had no influence on arterial stiffness or C-IMT measurements, or on central and peripheral blood pressure values, in subjects with some cardiovascular risk factors.

Vlachopoulos et al. [7], found that regular cocoa intake is related to decreased aortic stiffness and improved central hemodynamics. In their study population, the mean cocoa intake in healthy individuals was 4.63 g/day. Similar amount in terms of cocoa-intake to those obtained in our study (4.30 g /day). The inconsistency between this study and our findings merits comments. While subjects in the previous trial were healthy, our study included subjects with some cardiovascular risk factor (hypertension, diabetes, or dyslipidemia). These risk factors can influence the measures used to estimate arterial stiffness. In addition, many of the patients were receiving antihypertensive medication and lipid-lowering drugs, which may influence the results obtained. However, the interest of the study is that no previous evaluations have been made of the relationship between cocoa consumption and arterial stiffness in subjects of this kind.

However, data found in our population were similar to those collected by Ried K et al. [6], who concluded that cocoa was superior to placebo for reducing blood pressure in prehypertension. In the van den Bogaard B et al. study [8], cocoa increased 24-hour mean blood pressure and PWV, but decreased central systolic blood pressure and augmentation index. Thus, these authors did not also find a consistent relationship between cocoa intake and blood pressure and arterial stiffness measurements.

Djousse L and Lewis JR [9,10], recorded a inversely association between cocoa consumption and carotid atherosclerotic plaque prevalence. However, in coincidence of our study, no significant relationship was found between C-IMT and cocoa intake. There are several possible explanations for this results. In our study, the three groups of cocoa-intake are very heterogenous with differences in age, gender and the proportion of subjects with antihypertensive and lipid-lowering drugs. This fact may influence the results.

A limitation of the study is the statistical power because may not be sufficient to detect differences between cocoa-intake groups that could really exist in the population.

## Conclusions

In conclusion, in subjects with some cardiovascular risk factors, cocoa consumption does not imply improvement in the arterial stiffness values. However, further studies specifically designed to test the relationship are needed.

## Abbreviations

CBP: Central blood pressure; C-IMT: Intima-media thickness of the common carotid artery; PWV: Pulse wave velocity; CAIx: Central augmentation index; PAIx: Peripheral augmentation index; AASI: Ambulatory arterial stiffness index; ABI: Ankle-brachial index.

## Competing interests

The authors declare that they have no competing interests.

## Authors' contributions

JIR: devised the study, designed the protocol, participated in fund raising, interpretation of results, prepared the manuscript draft and corrected the final version of the manuscript. MAG and ER participated in the study design, interpretation of results, and manuscript review. CA participated in the study design, data collection and manuscript review. MCP performed all analytical methods, interpretation of results, and manuscript review. LG participated in the protocol design, fund raising, analysis of results, and final review of the manuscript. Finally, all authors reviewed and approved the final version of the manuscript.

## Authors' information

JIR (Nurse and dietitian). Member of the cardiovascular research group (RETICS RD06/0018/0027, REDIAPP) Carlos III Institute of Health of the Spanish Ministry of Health, SACYL. MAG and LG are MD, associate professors at the Department of Medicine, University of Salamanca and members of the cardiovascular research group (RETICS RD06/0018/0027, REDIAPP) Carlos III Institute of Health of the Spanish Ministry of Health, SACYL. CA (nurse) and ER (MD) are members of the cardiovascular research group (RETICS RD06/0018/0027, REDIAPP) Carlos III Institute of Health of the Spanish Ministry of Health, SACYL. MCP (PhD) is an Assistant professor at the Statistics Department, University of Salamanca.
